# Successful management of lymphangioma circumscriptum of the tongue with sirolimus monotherapy: a case report

**DOI:** 10.1007/s10006-026-01604-x

**Published:** 2026-07-27

**Authors:** Allison Salmon, Paige Reilly, Ashleigh Weyh, Nick Callahan

**Affiliations:** 1 UIC College of Dentistry , 801 South Paulina Street, Chicago, IL 60612 USA; 2 Rush University Hospital , 1620 W Harrison St, Chicago, IL 60612 USA

**Keywords:** Sirolimus, Rapamycin, Lingual lymphangioma, Congenital malformation, Nonsurgical treatment

## Abstract

Lymphangiomas are uncommon congenital benign tumors of the lymphatic system. They are typically diagnosed at birth and develop during the first years of life. The tongue is the most commonly affected structure in the oral cavity. Lymphangioma circumscriptum of the tongue is a common cause of macroglossia in children, and this macroglossia can lead to complications such as exclusive nasal breathing, airway obstructions, impaired oral feeding, esthetic disfigurement, and difficulties in mastication, swallowing, and speech. This report discusses the successful treatment of lymphangioma circumscriptum of the tongue with sirolimus, an immunosuppressant drug. The extent of the lesion made traditional surgical management impossible without excessive morbidity, so the patient was managed medically. This case report was unique due to its entirely nonsurgical treatment approach. The patient completed a 17-month course of sirolimus monotherapy with response to treatment monitored using T2 MRI. There was excellent response to treatment, full resolution of symptoms, and no treatment-related toxicity.

## Introduction

Lymphangiomas are uncommon congenital benign tumors of the lymphatic system. They are commonly present at birth and are typically diagnosed in the first two years of life, however they may go unnoticed until the eruption of the dentition or puberty [1, 2]. Few adult cases of lymphangiomas are reported. These lesions can cause macroglossia in children, complications of which include exclusive nasal breathing, airway obstruction, impaired feeding, esthetic disfigurement, and difficulties in mastication, swallowing, and speech [3].

There are two prominent theories on the formation of the lymphatic system. The centrifugal theory describes two endothelial buds emerging from the jugular sacs to develop into lymph vessels. In the centripetal theory, an anastomotically developing lymphatic system develops independently and eventually joins up to the central venous system. Regardless of which is the true explanation, a lymphangioma occurs when part of the lymphatic network fails to connect into the central network. This results in a build-up of lymph and an excessive expansion of an area of lymphatic vessels.

Lymphangiomas are classified by clinical presentation into three categories: macrocystic (cavities larger than 2 cm^3^), microcystic (cavities smaller than 2 cm^3^), and mixed (lesion with a combination of these two types) [4]. Three fourths occur in the head and neck region, most commonly on the dorsal tongue, then the lips, buccal mucosa, soft palate, and floor of mouth [5]. The next most affected areas are axilla and abdomen [6]. Lymphangiomas of the tongue are almost exclusively microcystic, and these lesions are referred to as ‘lymphangioma circumscriptum of the tongue.’

Clinical appearance varies based on the size and location of the lesion. Superficial lesions resemble pebbly, elevated, vesicle-like nodules with pink or yellow color, while deeper lesions appear as soft, diffuse nodular masses with more normal tissue color [7].

Treatment options for lymphangioma circumscriptum of the tongue have historically included surgery, injection of sclerosing agents, and low radiofrequency ablation. These lesions present specific therapeutic problems due to their microcystic character, how diffuse they can be, the functional impairments that would result from surgical removal, and high rate of recurrence. Complete surgical excision of these lesions is usually impossible due to the unacceptable degree of tissue resection it would require. A partial glossectomy has a high rate of relapse, often necessitates significant morphological modification of the tongue, and has painful, prolonged post-operative healing.

This clinical report describes a six year old patient with a symptomatic lymphangioma circumscriptum of the tongue. This case report was unique due to its entirely nonsurgical approach and adds to the growing literature of successful medical management of lymphangioma circumscriptum. This is the first case report to report sirolimus monotherapy as an effective treatment approach for an oral lymphangioma with minimal toxicity. 

## Case report

A six-year-old male patient was referred to the Oral & Maxillofacial Surgery (OMFS) Clinic for complete oral rehabilitation and biopsy of a right-sided tongue mass. The parents first noticed right tongue swelling when the patient was two, which progressively enlarged in size. At age five, the patient experienced an episode of acute dyspnea secondary to tongue swelling with associated tongue pain and was brought for emergency medical attention. He was treated for presumed anaphylaxis and received steroids. Subsequently, the patient had several more episodes of acute tongue swelling and pain. He experienced partial response of symptoms with steroid treatment.

**Fig. 1:**
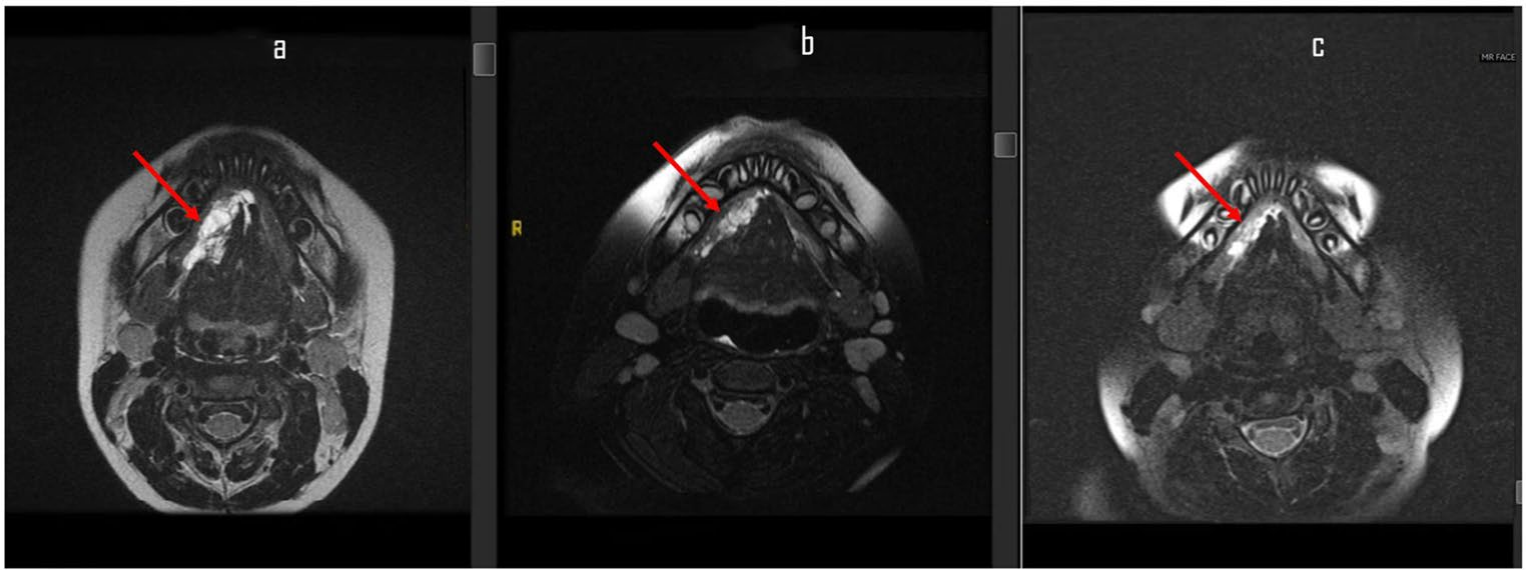
**a**, **b**, **c**: MRI Axial T2 at Diagnosis, after Six Months into Therapy, and 6 Months Post Completion of Therapy

A T2 MRI face was obtained and revealed an ill-defined microcystic lesion most consistent with a veno-lymphatic malformation of the right aspect of the tongue extending to the floor of the mouth (Fig. 1). Following the MRI, the patient underwent biopsy of the lesion with OMFS. Final pathology confirmed the diagnosis of lymphangioma circumscriptum.

Given the extent of lingual and oral floor involvement seen on T2 MRI, significant swelling on physical exam, and history of pain and acute respiratory compromise, surgical resection was deemed unfeasible without substantial morbidity. The patient was referred to Pediatric Hematology-Oncology clinic at Rush University Hospital for medical management. A trial of sirolimus, also known as rapamycin, was recommended based on published reports of producing favorable responses in treating lymphangiomas and other venous malformations. Sirolimus is an mTOR inhibitor immunosuppressant drug that inhibits the proliferation of and causes atrophy of lymphatic tissue. T2 MRI was used to assess response to treatment. The most significant adverse effect of sirolimus is its myelosuppressive activity, which can increase the risk of contracting pneumocystis pneumonia. To reduce this risk, prophylactic trimethoprim-sulfamethoxazole was recommended to be taken concurrently with the sirolimus therapy.

Potential airway compromise was the most significant factor impacting the decision to treat with sirolimus therapy. The clinical team were never able to verify if the patient’s episodes of respiratory distress were directly related to the tongue mass, however it was strongly suspected that it was a contributing factor. Further, there was concern that if the mass continued to progress, the patient would be placed at significant airway risk.

The Pediatric Hematology-Oncology team decided to begin treatment with sirolimus therapy, as it carried far greater benefit in terms of airway protection than any potential risks from treatment.

At one month follow up post diagnosis, in December of 2021, the patient’s baseline laboratory evaluation was unremarkable, and he was started on Sirolimus at oral doses of 0.8 mg/m^2^ every twelve hours with a goal trough level of 6–10 ng/mL. Due to the myelosuppressive effects of sirolimus, he was also prescribed trimethoprim-sulfamethoxazole to take on consecutive days of the week for pneumocystis pneumonia prophylaxis. The sirolimus therapy was delivered in an outpatient fashion with close monitoring of trough levels and CBC for adverse effects.

At follow up three weeks on therapy, the patient reported that his tongue “felt better” with no further episodes of pain. At follow up after two months on therapy, the patient and parent reported the tongue was noticeably decreased in size. Additionally, the patient’s nighttime snoring had resolved.

**Fig. 2:**
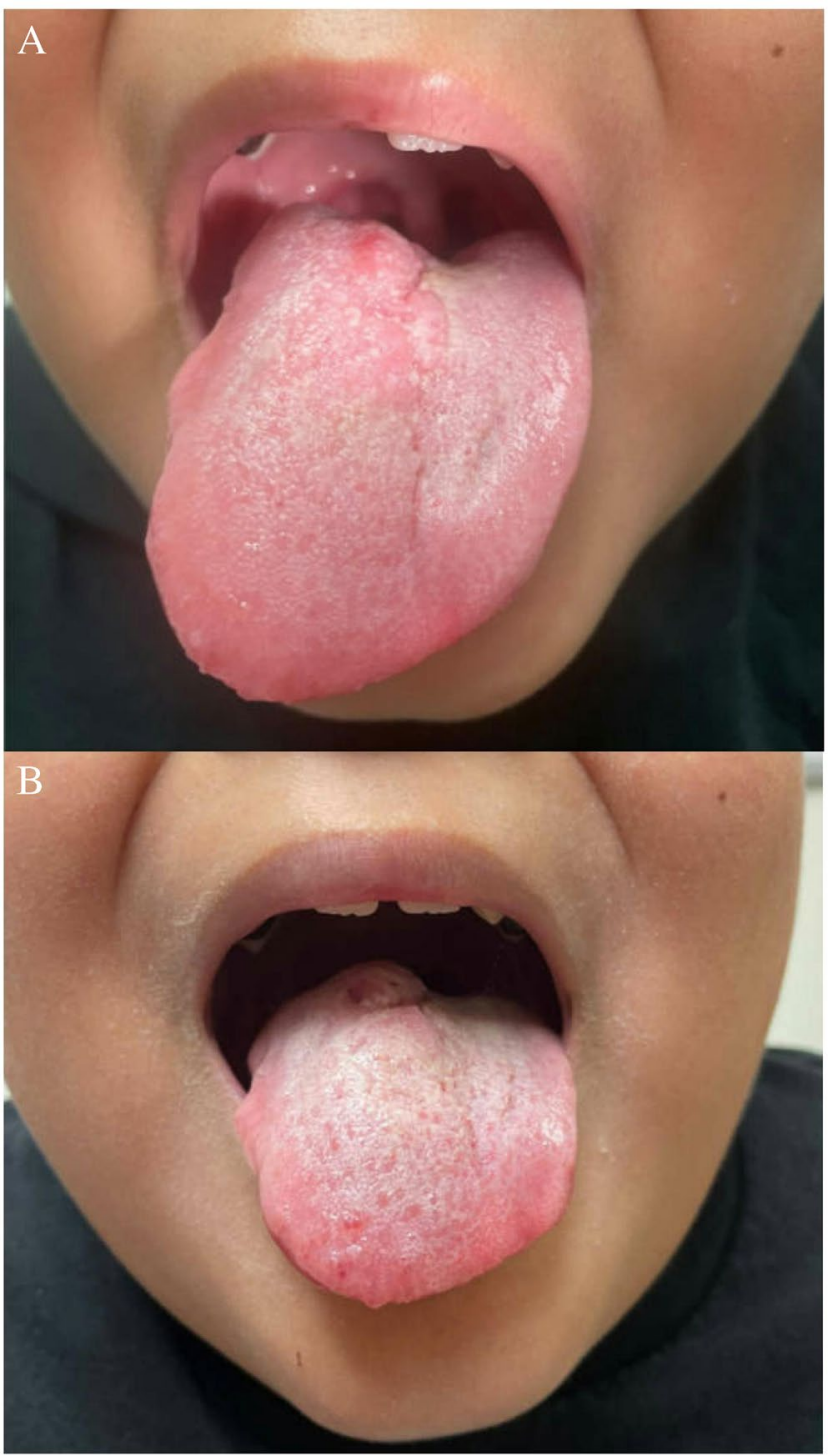
a and b: Clinical Photos Eight Months into Therapy and Eight Months Post Completion of Therapy

The patient continued to improve clinically over the course of therapy, with full resolution of intermittent tongue pain and no episodes of respiratory distress. T2 MRI imaging taken at diagnosis, six months into therapy, and six months after completion of therapy demonstrated significant improvement in lesion size (Fig. 1). Lymphangiomas are radiopaque on T2 MRIs due to their high water content, which allowed for good visualization of the lesion change over time. Clinical images of the tongue eight months into therapy versus eight months after completion of therapy show significant reduction in apparent hypertrophy of the posterior right tongue and improved symmetry of the posterior tongue (Fig. 2).

After close to seventeen months on therapy, sirolimus was discontinued. At follow up three months off therapy, the patient’s physical examination was unchanged, and he remained asymptomatic. At six months off therapy, follow up MRI demonstrated ongoing favorable response to treatment with no new mass or abnormal enhancement (Fig. 1). To date in February 2026, over four years after he began sirolimus treatment in December 2021, the patient has had no relapse of the lesion or of any symptoms related to the lesion.

## Discussion

In this case, we treated a patient’s lingual lymphangioma with sirolimus. This case is unique due to the treatment’s nonsurgical approach with sirolimus monotherapy. Sirolimus treatment has been reported in many case series of patients with lymphangiomas and other venous malformations with 80–90% partial responses. The toxicity profile is favorable, but it is myelosuppressive [2]. Sirolimus treatment has been associated with opportunistic infections as well as hyperlipidemia. Of the lymphatic malformation patients in the 2018 Wiegand systematic review, thirty-five had partial responses, one showed no response, two had progressive disease, and one had an unevaluable outcome. The systematic review concluded sirolimus therapy may be effective for lymphatic malformations, but stipulated that further randomized controlled studies are required for clarity [8].

**Fig. 3:**
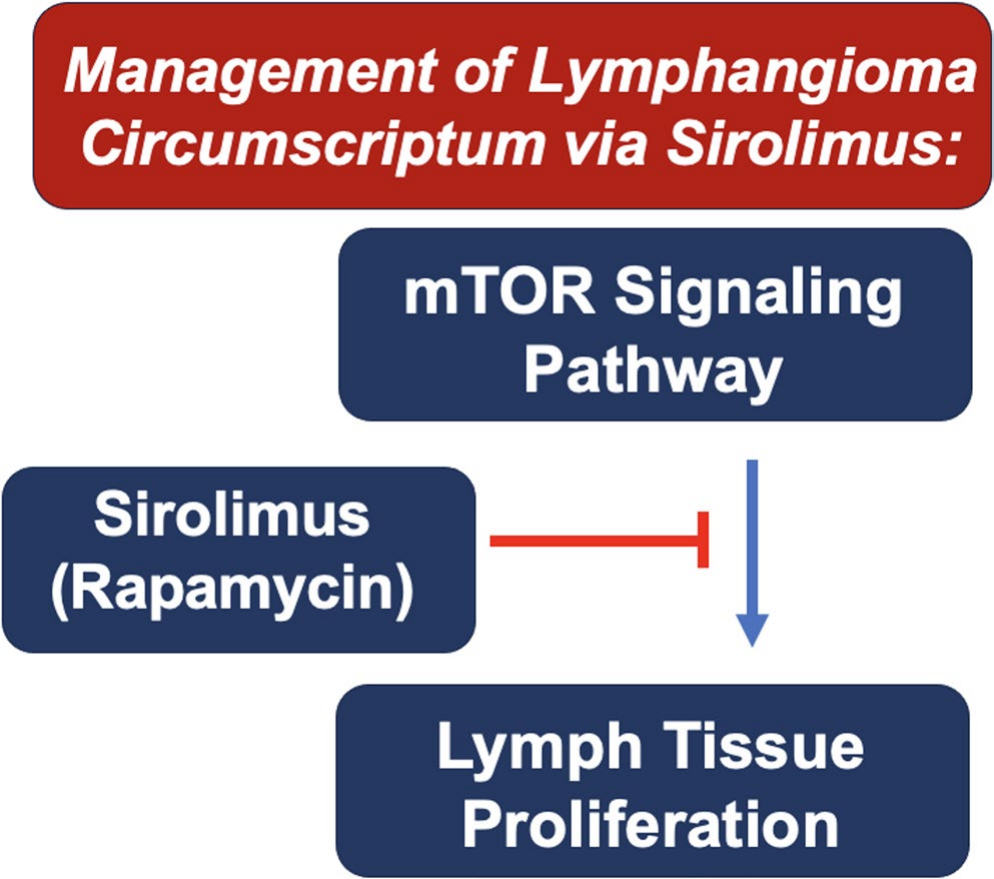
Sirolimus Mechanism of Action Diagram

Sirolimus is an mTOR inhibitor that targets the PI3K-AKT-mTOR signaling pathway. The mTOR pathway is involved in the control of lymphatic endothelial and valvular morphogenesis. The exact mechanisms of this pathway have yet to be fully characterized. Inhibition of the mTOR pathway ultimately results in the suppression of lymphangiogenesis as well as the suppression of the T-cell immune response. This relationship is illustrated in Fig. 3. Treatment with sirolimus causes atrophy in lymphangioma lesions and decreases their growth [9]. A 2015 clinical trial of sirolimus followed up with fifteen patients twelve months post treatment. They found that all patients with lymphatic malformations experienced almost complete relief of pain and symptoms, improved functional restraint, and self-perceived quality of life. Side effects were principally mucositis, mild headache, fatigue, and diarrhea. A statistically significant reduction in volume was observed, with MRIs showing a decrease in most patients that reached one-year follow-up [10].

Other treatment options for lymphangioma circumscriptum of the tongue include surgery, injection of sclerosing agents, and low radiofrequency ablation [5]. Complete surgical excision was not feasible in this case due to the need for radical resection and the resulting loss of structure and function, in addition to the painful, prolonged post-operative healing that would result. Furthermore, treatment via surgery has a high rate of relapse. The injection of sclerosing agents is not effective with microcystic lesions, making it a poor treatment option. Low radiofrequency treatment, also known as ‘coblation’ or ‘controlled ablation,' involves the local delivery of low radiofrequency energy via an electrode and a conducting medium to induce local tissue destruction and create a secondary fibrosis. This was a viable treatment option, however previous case studies have shown that the treatment is not curative, and that regrowth, aggravation of the lesion, and recurrence are all possible after the conclusion of treatment [4]. With the various treatment options in mind, sirolimus monotherapy was selected due to being a less invasive treatment option with a high potential for success.

The primary goal of treatment in this case was a full resolution of intermittent tongue pain, no further episodes of respiratory distress, and no treatment related toxicity, which were all fully achieved. After three weeks of sirolimus therapy and trimethoprim-sulfamethoxazole prophylaxis, the patient reported no further episodes of pain. Sirolimus therapy was discontinued after seventeen months of therapy. The six-month MRI demonstrated ongoing favorable response to treatment with no new mass or abnormal developments. To date in May 2025, nearly four years after he began sirolimus treatment in December 2021, the patient has had no relapse of the lesion or of any symptoms related to the lesion.

The sirolimus therapy in this case was started outpatient with close monitoring of trough levels and CBC for adverse effects, which is the typical fashion for this drug. The effects of sirolimus have a very slow onset, so the benefits of inpatient initiation of treatment are limited as there was no immediate effect to monitor.

A limitation of this report is that it only follows one case, so further research is necessary to confirm the efficacy and safety of sirolimus monotherapy in a larger patient population. Furthermore, continued follow up with this patient in the future would be necessary to verify the durability of the treatment effect.

## Conclusion

This case adds to the growing body of evidence that demonstrates the effectiveness of sirolimus monotherapy in the management of lingual lymphangiomas, particularly when surgical resection is not feasible. Sirolimus has a favorable toxicity profile with its myelosuppressive effects as a main disadvantage. Partial responses with 80–90% effectiveness have been reported [2].

In the future, sirolimus monotherapy has the potential to become a more common first-line treatment modality for lymphatic malformations, as it can lead to less complications than surgery and other treatment modalities. It could also be utilized in the future as a preparatory measure to decrease lesion size prior to surgery, if surgical intervention is necessary. Further research is necessary to determine the longevity of treatment outcomes and the potential broader applications of sirolimus in managing complex lymphatic malformations.

## Data Availability

No datasets were generated or analysed during the current study.
